# Development of Anti-VEGF Therapies for Intraocular Use: A Guide for Clinicians

**DOI:** 10.1155/2012/483034

**Published:** 2011-12-18

**Authors:** Pearse A. Keane, Srinivas R. Sadda

**Affiliations:** ^1^NIHR Biomedical Research Centre for Ophthalmology, Moorfields Eye Hospital NHS Foundation Trust and UCL Institute of Ophthalmology, London EC1V 2PD, UK; ^2^Doheny Eye Institute and Department of Ophthalmology, Keck School of Medicine, University of Southern California, Los Angeles, CA 90033, USA

## Abstract

Angiogenesis is the process by which new blood vessels form from existing vessel networks. In the past three decades, significant progress has been made in our understanding of angiogenesis; progress driven in large part by the increasing realization that blood vessel growth can promote or facilitate disease. By the early 1990s, it had become clear that the recently discovered “vascular endothelial growth factor” (VEGF) was a powerful mediator of angiogenesis. As a result, several groups targeted this molecule as a potential mediator of retinal ischemia-induced neovascularization in disorders such as diabetic retinopathy and retinal vein occlusion. Around this time, it also became clear that increased intraocular VEGF production was not limited to ischemic retinal diseases but was also a feature of choroidal vascular diseases such as neovascular age-related macular degeneration (AMD). Thus, a new therapeutic era emerged, utilizing VEGF blockade for the management of chorioretinal diseases characterized by vascular hyperpermeability and/or neovascularization. In this review, we provide a guide for clinicians on the development of anti-VEGF therapies for intraocular use.

## 1. Introduction

In 1948, Isaac Michaelson proposed that a diffusible factor (named afterward “factor X”) could be responsible, not only for the development of the normal retinal vasculature but also for pathological neovascularization in proliferative diabetic retinopathy and other ocular disorders [1]. By the early 1990s, it had become clear that the recently discovered “vascular endothelial growth factor” (VEGF) possessed many of the requisite characteristics of a “factor X” [2]. As a result, several groups targeted this molecule as a potential mediator of retinal ischemia-induced neovascularization in disorders such as diabetic retinopathy and retinal vein occlusion (RVO) [3, 4]. Around this time, it also became clear that increased intraocular VEGF production was not limited to ischemic retinal diseases but was also a feature of choroidal vascular diseases such as neovascular age-related macular degeneration (AMD) [5, 6]. Thus, a new therapeutic era emerged, utilizing VEGF blockade for the management of chorioretinal diseases characterized by vascular hyperpermeability and/or neovascularization.

In this review, we begin by providing an overview of angiogenesis, the manner in which VEGF was discovered to be central to this process, and then a summary of VEGF biology. In this manner, we aim to provide the clinician with an understanding of the clinical scenarios in which VEGF blockade is likely to be successful and of patient benefit. We continue by describing the development of four key anti-VEGF therapies (pegaptanib, bevacizumab, ranibizumab, and aflibercept) and the results of their application in a selection of pioneering clinical trials. By describing the main features of their development in a manner accessible to clinicians, we aim to highlight those molecular characteristics, of each agent, with implications for clinical outcomes and patient safety. We conclude the review by describing likely future directions in the application of anti-VEGF therapy in chorioretinal disease.

## 2. Angiogenesis

### 2.1. Overview

Angiogenesis is the process by which new blood vessels form from existing vessel networks (by comparison, vasculogenesis is a form of de novo blood vessel formation that is typically seen in the embryo) [7–9]. Angiogenesis begins with vasodilatation and increases in vascular permeability, followed by activation and proliferation of vascular endothelial cells; these changes are accompanied by degradation of the surrounding extracellular matrix (ECM), facilitating endothelial cell migration. The migrating endothelial cells assemble, form cords, and ultimately acquire lumens; further differentiation to accommodate local requirements then occurs and a network of similarly differentiated periendothelial cells and matrix develops. After further remodeling a complex vascular network is ultimately formed.

### 2.2. Role of Angiogenesis in Disease

In the past three decades, significant progress has been made in our understanding of angiogenesis: progress driven in large part by the increasing realization that blood vessel growth can promote or facilitate disease [10]. This major conceptual advance first occurred in the 1930s and 1940s, when it was hypothesized that induction of blood vessel growth through release of vasoproliferative factors would confer a growth advantage on tumor cells [11]. Subsequently, in the 1970s, Folkman hypothesized that blockade of angiogenesis could be a strategy to treat cancer and other disorders [12]. However, adoption of such a strategy first required the identification and characterization of the mediators of angiogenesis—a major technological challenge at that point.

### 2.3. Putative Regulators of Angiogenesis

In the subsequent years, advances in molecular biology led to the identification of many putative regulators of angiogenesis, with well-known examples including basic fibroblast growth factor (bFGF), transforming growth factor (TGF)-*β*, and the angiopoietins [7]. In the 1980s, bFGF was thought to be the major angiogenic factor in the pituitary and other organs. However, this model was called into question when, in 1986, it became clear that bFGF lacks a peptide sequence necessary for secretion and is thus confined intracellularly (angiogenesis is a process that requires diffusion in an extracellular environment) [13].

### 2.4. Discovery of VEGF

In the mid-1980s, Ferrara and Henzel cultured a population of nonhormone secreting follicular cells—with unusual characteristics—from bovine pituitary glands (follicular cells have cytoplasmic projections that establish intimate connections with perivascular spaces and were thought to have a role in regulating growth and maintenance of pituitary vasculature) [14]. Ferrara discovered that culture medium conditioned by these cells strongly promoted endothelial cell growth. He hypothesized that this mitogenic activity may be the result of a secreted protein; the subsequent isolation and sequencing of this protein led to discovery of the most important mediator of angiogenesis currently known—VEGF [15].

### 2.5. Vascular Permeability Factor

Independently, in the early 1980s, Senger et al. had reported the identification of a permeability-enhancing protein (in the supernatant of a guinea pig tumor cell line), which they named “vascular permeability factor” (VPF) [16]. In 1989, at the same time Ferrara and coworkers were reported their discovery of VEGF. Keck et al. reported the isolation and sequencing of VPF [17]. Surprisingly, their findings indicated that VEGF and VPF were, in fact, the same molecule.

### 2.6. Clinical Role for VEGF Blockade

Although multiple growth factors other than VEGF have been implicated in the angiogenic process (e.g., bFGF), VEGF appears critical for a number of reasons: its production is driven by hypoxia; it is highly selective for endothelial cells, it possesses diffusion characteristics that allow it to reach its target, and it affects multiple aspects of the angiogenic process [18, 19]. VEGF also causes vascular dilatation and promotes vasopermeability, both of which facilitate a rich environment for the growth of new vessels. Thus, despite the complexity of the angiogenic process, and the potential redundancy of the growth factors involved, VEGF blockade was quickly recognized as a promising approach for the restriction of blood vessel formation in a variety of pathologic scenarios [8].

## 3. VEGF Biology

### 3.1. Gene Family

VEGF-A, first discovered in 1989 (see above), is the prototype member of a gene family (i.e., a group of genes with shared sequences and with similar biochemical functions) that also includes placental growth factor (PLGF), VEGF-B, VEGF-C, VEGF-D, and VEGF-E (prior to the discovery of other family members, VEGF-A was known simply as VEGF; the terms are used interchangeably in this review) [10, 18]. Of note, VEGF-C and VEGF-D are involved in the regulation of lymphatic angiogenesis [20], demonstrating the unique role of this gene family in controlling multiple structural components of the vascular system.

### 3.2. Regulation of VEGF Gene Expression

Oxygen tension has a key role in regulating the production of VEGF. VEGF mRNA expression is induced by exposure to low oxygen tension under a variety of pathophysiological circumstances, and it is now well established that a transcription factor, hypoxia-inducible factor-1 (HIF-1), is a key mediator of this response [21, 22]. Recent studies have also shown that Von Hippel Lindau (VHL) protein, a product of the VHL tumor suppressor gene, provides negative regulation of VEGF and other hypoxia-inducible genes (inactivation of this gene leads to development of capillary hemangioblastomas in the retina and cerebellum, and in many cases, renal cell carcinomas) [23]. 

Several major growth factors, such as epidermal growth factor, also upregulate VEGF mRNA expression, suggesting that paracrine or autocrine release of such factors works in concert with local hypoxia to increase production of VEGF [24, 25]. In addition, inflammatory cytokines, such as interleukin-1*α* and interleukin-6, induce expression of VEGF in several cell types (an observation in agreement with the hypothesis that VEGF plays a role in the angiogenesis and hyperpermeability seen in some inflammatory disorders) [25].

### 3.3. VEGF Isoforms

The human *VEGFA *gene is organized as eight exons separated by seven introns (i.e., eight expressed regions that are joined together in the final mature RNA) [26]. Alternative splicing of the *VEGFA *gene results in the generation of four major isoforms (VEGF_121_, VEGF_165_, VEGF_189_, and VEGF_206_), having, respectively 121, 165, 189, and 206 amino acids. VEGF_165_ is the predominant isoform [27]. 

Native VEGF is a heparin-binding glycoprotein (heparin is commonly used during protein purification due to its structural similarity to RNA and DNA), with a protein molecular weight of 45 kDa, the properties of which correspond closely to those of VEGF_165_ [27]. Loss of the heparin-binding domain of VEGF results in a significant loss in its mitogenic activity [28]. VEGF_121_, while freely diffusible in the ECM, is acidic and does not bind heparin [27]. Conversely, VEGF_189_ and VEGF_206_, while being highly basic and capable of binding heparin with high affinity, are almost completely sequestered in the ECM. Thus, VEGF_165_, with intermediary properties, possesses the optimal characteristics of bioavailability and biological potency [27].

### 3.4. VEGF Receptors

VEGF binds to two, related, receptor tyrosine kinases: VEGF Receptor 1 (VEGFR1) and VEGF Receptor 2 (VEGFR2) [27]. Both VEGFR1 and VEGFR2 have seven immunoglobulin-like domains in the ECM, a single transmembrane region, and a tyrosine kinase sequence interrupted by a kinase-insert domain. In 1992, VEGFR1 was the first VEGF receptor discovered and was found to bind VEGF with high affinity [29]. However, despite its lower binding affinity for VEGF relative to VEGFR1, there is now agreement that VEGFR2 is the major mediator of the mitogenic, angiogenic, and permeability-enhancing effects of VEGF (the precise function of VEGFR1 is still under debate but may provide a “decoy effect” on VEGF signaling) [27]. In addition, VEGF interacts with a family of nonsignaling coreceptors, the neuropilins—neuropilin-1 (NRP-1) appears to present VEGF_165_ to VEGFR2 in a configuration that increases the effectiveness of VEGFR2-mediated signal transduction [18, 30].

### 3.5. Activities of VEGF

Vascular endothelial cells are the primary targets for VEGF biologic activity, with their mitogenic effects well documented, both *in vitro* and* in vivo* [27]. In particular VEGF induces a potent angiogenic effect in a variety of animal models *in vivo* [15, 31].

VEGF also acts as a survival factor for endothelial cells in a variety of circumstances. Inhibition of VEGF results in extensive apoptotic changes in the vasculature of neonatal, but not adult mice [32]; furthermore, a marked VEGF dependence has been demonstrated in the endothelial cells of newly formed but not of established vessels within tumors [33]. Coverage by pericytes is thought to be one of the key events, resulting in loss of VEGF dependence [34]. 

VEGF has also been shown to act as a chemotactic agent for bone marrow-derived monocytes [35], a pro-inflammatory cytokine through upregulation of intercellular adhesion molecule-1 (ICAM-1) with consequent leukocyte adhesion [36], and a promoter of blood vessel extravasation through the upregulation of matrix metalloproteinases and decreased release of metalloproteinase inhibitors [37]. 

The effects of VEGF on the promotion of vascular leakage, both in inflammation and in other pathologic circumstances, are also well established (prior to its isolation and sequencing, VEGF was initially characterized as “vascular permeability factor” by Senger et al. (see above)) [16]. Consistent with this role, VEGF has been shown to promote dissolution of tight junctions between endothelial cells and to induce endothelial fenestration in a number of vascular beds [38]. VEGF also induces vasodilatation in a dose-dependent fashion as a result of release of endothelial cell-derived nitric oxide—systemic blockade of VEGF may thus result in a clinically significant adverse hypertensive effect [39]. 

Taken together, blockade of the biologic effects of VEGF results in rapid vessel remodeling with regression of pericyte-poor capillaries, reductions in vascular lumen diameter, and reductions in vascular permeability [33, 34]. More recently, evidence has suggested that VEGF could have additional neuroprotective effects [40].

### 3.6. Role of VEGF in Ocular Disease

In 1994, Aiello et al. found a striking correlation between intraocular VEGF concentrations and active proliferative retinopathy in patients with diabetes and ischemic central retinal vein occlusion (CRVO) [3]. Around the same time, Adamis et al. reported increased concentrations of VEGF in the vitreous of patients with diabetic retinopathy [4]. In 1996, it also became clear that increased intraocular levels of VEGF were not limited to ischemic retinal disorders: in a pair of influential studies, the localization of VEGF to choroidal neovascular membranes in patients with neovascular AMD was reported [5, 6]. Proof-of-concept studies then demonstrated that blockade of VEGF, in animal models, led to marked decreases in retinal and iris neovascularization [41, 42]. Furthermore, exogenous administration of VEGF was demonstrated to produce retinal ischemia and vascular hyperpermeability in primates [43].

## 4. Pegaptanib

Pegaptanib sodium is an RNA aptamer that binds to the heparin-binding domain of VEGF and, thus, prevents the predominant VEGF_165_ isoform from binding to VEGF receptors [44]. Pegaptanib was licensed to EyeTech Pharmaceuticals (now OSI Pharmaceuticals) for late stage development and marketing in the United States as “Macugen” (outside the USA, pegaptanib is marketed by Pfizer Inc.).

### 4.1. Chemistry

Aptamers (from the Latin *aptus, *to fit, and the Greek *meros, *part or region) are oligonucleotides that bind to specific target molecules and that are usually created by selection from a large random sequence pool [45]. In this manner, aptamers are commonly used for basic research and clinical purposes as macromolecular drugs. Aptamers constitute one of four classes of oligonucleotide reagents, the others being antisense oligonucleotides, ribozymes, and small interfering RNAs (siRNAs) [44]. However, in contrast with these other entities, aptamers can act on extracellular targets and, therefore, are not required to cross cell membranes to exert their therapeutic effects.

The selection of aptamers has become relatively straightforward with the advent of “systematic evolution of ligands by exponential enrichment” (SELEX) [46]; in this process, aptamers are engineered to bind to various target molecules through repeated rounds of *in vitro* selection. Aptamers offer molecular recognition properties that rival that of antibodies, but with a number of advantages: (1) they can be engineered completely *in vitro*, (2) they are readily produced by chemical synthesis, (3) they possess desirable storage properties, and (4) they elicit little or no immunogenicity [44, 45]. Pegaptanib has the distinction of being the first aptamer therapeutic approved for use in humans [44]. 

Having chosen VEGF_165_ as the target for selection of a prospective anti-VEGF aptamer, three separate iterations of the SELEX methodology were carried out by scientists at NeXstar Pharmaceuticals [44]. By 1998, three, stable, high-affinity anti-VEGF_165_ aptamers had been characterized, one of which was selected for development as pegaptanib (initially designated NX1838, and then, EYE001) (all three aptamers demonstrated little or no binding to VEGF_121_) [47].

### 4.2. Preclinical Studies

The fact that pegaptanib offers selective inhibition of a single isoform offers the theoretical advantage that “normal” vessels may be maintained by VEGF_121_ and other isoforms, while pathologic neovascularization may be suppressed [18, 44, 48]. Indeed, prior to clinical trials in humans, basic research demonstrated that administration of EYE001 (pegaptanib) could lead to both reduced vascular permeability and inhibition of both corneal and retinal neovascularization [49]. It has subsequently been shown, however, that various proteases activated during angiogenesis may cleave VEGF_165_ (and longer isoforms) to generate nonheparin binding fragments—such fragments may be sufficient to drive angiogenesis while evading pegaptanib blockade [50, 51].

### 4.3. Pharmacokinetics and Metabolism

Nonmodified aptamers are rapidly cleared from the body, with a half-life of minutes to hours, as a result of nuclease degradation and renal clearance (a result of the inherently low molecular weight of aptamers). Therefore, modification of aptamers, such as 2′-fluorine-substituted pyrimidines, and polyethylene glycol (PEG) linkage, can be used to increase their stability and terminal half-life (both approaches are used in the case of pegaptanib) [44]. Using these approaches pegaptanib has been found to be stable in human plasma, at ambient temperatures, for more than 18 hours [52]. 

Pegaptanib pharmacokinetics have been evaluated following intravitreal injection in monkeys and rabbits [49, 52, 53]. In both animal models, pegaptanib was detected in the vitreous at biologically active levels for at least 28 days following a single 0.5 mg intravitreal injection. In rabbits, after a single dose of pegaptanib, the initial vitreous humor levels were approximately 350 *μ*g/mL and decreased by an apparent first-order elimination process to approximately 1.7 *μ*g/mL by day 28. By comparison, the plasma concentrations of pegaptanib were significantly lower, ranging from 0.092 *μ*g/mL to 0.005 *μ*g/mL (day 1 to day 21). Plasma levels also declined by an apparent first-order elimination. In a human pharmacokinetic study, pegaptanib was not found to accumulate in the plasma after multiple doses (i.e., systemic exposures were similar at different time-points); furthermore, no antipegaptanib antibodies (IgG or IgM) were detected [54].

### 4.4. Selected Clinical Studies: Neovascular AMD

In 2004, following publication of results from two, concurrent, phase III clinical trials (the VEGF Inhibition Study in Ocular Neovascularization, or VISION, trials), pegaptanib was licensed for use in the USA by the Food and Drug Administration (FDA) [55]. The VISION trials—two large-scale, multicenter, randomized, controlled, clinical trials—demonstrated that intravitreal administration of 0.3 mg of pegaptanib at six weekly intervals, for a period of 48 weeks (a total of nine treatments), was effective in reducing moderate vision loss in patients with neovascular AMD (higher doses were not shown to provide clinical benefit). In these studies, 70% of pegaptanib-treated patients avoided further moderate visual loss (defined in most AMD studies as a loss of fewer than 15 letters of visual acuity) compared with 55% of sham-treated patients. However, treated eyes still lost, on average, 1.5 lines of visual acuity over the course of a year of treatment. There was no evidence of either systemic toxicity or an increased risk of potential VEGF inhibition-related adverse events (a safety profile confirmed following three years of treatment/follow-up) [56].

### 4.5. Selected Clinical Studies: Diabetic Macular Edema

In 2011, the results of a phase II/III-randomized controlled trial, of intravitreal pegaptanib for the treatment of diabetic macular edema (DME), were published [57]. In this study, subjects with DME received injections of 0.3 mg of intravitreal pegaptanib, or sham injections, every six weeks for a year, and then according to prespecified criteria in a second year. In all, 36.8% of patients receiving pegaptanib, versus 19.7% of those in the sham group, experienced an improvement in visual acuity greater than 10 letters when compared to baseline. After two years, pegaptanib-treated patients gained, on average, 6.1 letters of visual acuity (versus 1.3 letters for controls). Pegaptanib-treated patients also received fewer focal/grid laser treatments (subjects were eligible for this beginning at week 18).

## 5. Bevacizumab

Bevacizumab (Avastin, Genentech, South San Francisco, CA) is a full-length monoclonal antibody, first derived from a murine source and prepared for intravenous administration, which binds to and inhibits all isoforms of VEGF [18, 58]. Bevacizumab was originally developed and approved for the treatment of metastatic colorectal cancer but may also be of benefit in the treatment of nonsmall cell lung cancer, metastatic breast cancer, and glioblastoma multiforme [59]. Use of bevacizumab in these contexts has been associated with increased incidences of hypertension, bleeding, and thromboembolic events [59]. However the doses employed for intraocular use are many times lower than those used systemically, and the efficacy and safety of bevacizumab, for the treatment of neovascular AMD, has recently been demonstrated in phase III clinical trials [60, 61].

### 5.1. Chemistry

Bevacizumab was originally developed from a mouse antihuman VEGF antibody (A.4.6.1), generated from mice immunized with the VEGF_165_ isoform [58]. A.4.6.1 recognizes all isoforms of VEGF and, in 1992, was shown to inhibit growth of human tumor cell lines *in vivo* [62]. Subsequently, in 1996, intraocular administration of A.4.6.1 was found to inhibit iris neovascularization occurring secondary to retinal ischaemia in a primate model [42]. In 1997, bevacizumab was developed by humanization of A.4.6.1 [63]. In this process, six complementarity-determining regions (CDRs) (i.e., regions that determine antibody-binding) were transferred from A.4.6.1 to a human antibody framework previously used for humanizations. However, this transfer reduced VEGF binding over 1000 fold—to reduce this effect, eight framework residues were changed from human to murine.

Bevacizumab is produced in Chinese hamster ovary cells using expression plasmids (plasmids are DNA molecules separate from chromosomal DNA that can be used to manufacture large quantities of proteins) [58]. Bevacizumab is a 149 kDa full-length antibody, composed of two light chains and two heavy chains, and with a 93% human amino acid sequence.

### 5.2. Preclinical Studies

The effects of bevacizumab have been examined in a number of *in vitro *and *in vivo *studies [58]; as bevacizumab was not developed with the intention of intraocular administration, many of these studies were performed only after its widespread adoption in this manner for clinical practice. In both murine and porcine models, bevacizumab has been demonstrated to reduce VEGF-induced permeability and proliferation of choroidal endothelial cells and to inhibit VEGF-induced migration of human umbilical vein endothelial cells [64–66]. In addition, bevacizumab has been demonstrated as nontoxic, or not to alter the viability of, neurosensory retinal cells, retinal ganglion cells, and human retinal pigment epithelium (RPE) cells [58]. Concern has also been raised about the Fc component present in full-length antibodies such as bevacizumab—Fc domains are known to initiate complement activation and immune cell destruction [18]. Recent studies have demonstrated that choroidal neovascular membranes from patients with neovascular AMD treated with bevacizumab are characterized by significantly higher inflammatory activity [67]. Preliminary results have also demonstrated that bevacizumab Fc domains are capable of binding effectively to human RPE and human umbilical vascular endothelial cell (HUVEC) membranes via Fc receptors, activating the complement cascade and leading to cell death [58].

### 5.3. Pharmacokinetics and Metabolism

Bevacizumab was developed for intravenous administration in diseases such as colorectal cancer [59]. As a result, compounding into smaller doses is required for intraocular administration. Studies have demonstrated differences in bevacizumab concentration and the presence of particulate contaminants following this process, emphasizing the need for implementation of optimal protocols when compounding pharmacies prepare this drug for intravitreal use [58, 68].

The pharmacokinetics of bevacizumab, following intravitreal administration, have not been well characterized. Knowledge of the vitreous half-life is an important consideration when optimizing retreatment frequencies, whereas serum concentrations are an important factor with respect to systemic adverse effects (e.g., stroke). In rabbits receiving 1.25 mg of bevacizumab, the vitreous half-life was 4.32 days (versus 2.88 days for ranibizumab), and the maximum serum concentrations were reached after eight days [69, 70]. Small amounts of bevacizumab were also detected in the vitreous of the fellow, uninjected eye. In a more recent study performed in humans, an aqueous half-life of 9.82 days was found after intravitreal injection of 1.5 mg of bevacizumab [71]. 

The retinal penetration of bevacizumab has also been studied in animal models (experience with retinal penetration of other, full-length antibodies suggested that their large size would act as a limiting factor). In rabbits, Shahar et al. demonstrated, using confocal immunohistochemistry, that full thickness retinal penetration occurred 24 hours after intravitreal injection; this study also demonstrated the essential absence of bevacizumab from the retina by four weeks post injection [72].

### 5.4. Selected Clinical Studies: Neovascular AMD

In 2010, the results of the ABC (Avastin (Bevacizumab) for treatment of Choroidal Neovascularization) trial provided the first evidence from a phase III-randomized controlled study for the efficacy of intravitreal bevacizumab in neovascular AMD [60, 73]. In this single year trial, 32% of patients treated with bevacizumab gained 15 or more letters from baseline visual acuity (initial three month loading phase, and then retreatment as required). In addition, 91% of patients receiving bevacizumab lost fewer than 15 letters of visual acuity from baseline, and mean visual acuity increased by 7.0 letters over the study period.

In 2011, the results of the CATT (Comparison of Age-Related Macular Degeneration Treatments Trials) study provided further evidence, from larger phase III trial, for the efficacy of bevacizumab in neovascular AMD [61]. In this trial, 31.3% of patients treated with bevacizumab on a fixed, monthly regimen gained 15 or more letters from baseline visual acuity (28.0% for patients treated with bevacizumab as required). In addition, 94.0% of patients receiving bevacizumab on a fixed, monthly regimen lost fewer than 15 letters of visual acuity from baseline (91.5% in the bevacizumab as required group). Finally, mean visual acuity increased by 8.0 letters over the study period in those receiving bevacizumab monthly (5.9 letters in bevacizumab as required group). Of note, statistical comparisons between bevacizumab given as needed, and given on a fixed, monthly regimen, were inconclusive.

## 6. Ranibizumab

Ranibizumab (formerly known as rhuFAb V2) is an antibody fragment that binds and inhibits all isoforms of VEGF [18]. Specific development of ranibizumab for intraocular use was driven, in part, by preliminary studies suggesting that full-length monoclonal antibodies would not distribute across all retinal layers [74]. Furthermore, the relatively long systemic half-life of full-length antibodies (versus antibody fragments) raised concerns about systemic toxicity in patients requiring long-term anti-VEGF blockade [18]. 

### 6.1. Chemistry

Bevacizumab is constructed from A.4.6.1 using one of 12 possible Fab (fragment antigen binding) variants: “Fab 12” [58]. Ranibizumab is constructed using a different Fab variant from A.4.6.1: “Fab MB1.6”, in an effort to obtain higher binding affinities for VEGF [75]. Ranibizumab is produced as a 48 kDa antibody fragment, in *E. coli*, using expression plasmids [58]. It is a chimeric molecule, consisting of an antigen-binding murine component, and a nonbinding human component that serves to make it less antigenic (in Greek mythology, the chimera was a monster with a lion's head, a goat's body, and a serpent's tail). On a molar basis, ranibizumab is between five- and 20-times more potent than bevacizumab at binding of VEGF [75].

### 6.2. Preclinical Studies

Preclinical studies have demonstrated the safety, tolerability, and efficacy of ranibizumab in animal models. In particular, intravitreal administration of ranibizumab reduced vascular leakage in a monkey model of choroidal neovascularization (CNV), while pretreatment with ranibizumab prevented laser-induced development of CNV in this model [76].

### 6.3. Pharmacokinetics and Metabolism

The pharmacokinetics of ranibizumab, after intravitreal administration, have been studied both in animal models and in human trials [69, 77, 78]. Ranibizumab is thought to exit the vitreous cavity posterior via retinal penetration and choroidal vascular drainage or anteriorly via the aqueous drainage route. In animal studies, ranibizumab is cleared from the vitreous with a half-life of approximately three days [69]. Therefore, ranibizumab is thought to maintain biologically active retinal concentrations for approximately one month. After reaching a maximum at approximately one day, the serum concentration of ranibizumab declines in parallel with this. In human studies, following monthly intravitreal ranibizumab administration, maximum serum concentrations were dose dependent but low (0.3 ng/mL to 2.36 ng/mL—levels more than 1000 fold lower than in the vitreous and thought to be below the concentrations necessary for reduction in biological activity of VEGF by 50%) (http://www.gene.com/). In a recent study by Bakri et al., no ranibizumab was detected in the serum, or the fellow uninjected eye, of rabbits injected with 0.5 mg of intravitreal ranibizumab; by comparison, small amounts of bevacizumab were detected, both in the serum and in the fellow-uninjected eye [69].

### 6.4. Selected Clinical Studies: Neovascular AMD

In 2006, ranibizumab was licensed for use in the United States following publication of the MARINA and ANCHOR studies [79, 80].

In the MARINA (Minimally Classic/Occult Trial of the Anti-VEGF Antibody Ranibizumab in the Treatment of Neovascular AMD) trial, patients with either “minimally classic” or “occult” angiographic leakage patterns were randomized to receive monthly injections of intravitreal ranibizumab (0.3 mg or 0.5 mg) or monthly sham injections [80]. At the 12-month point of this study, visual acuity had improved by 15 or more letters in 33.8% of the 0.5 mg group (as compared with only 5.0% of the sham-injection group). Furthermore, patients receiving ranibizumab, on average, demonstrated increases in visual acuity (7.2 letters in the 0.5 mg group), while those receiving sham therapy, on average, demonstrated losses (10.4 letters in the sham-injection group). In addition, the vast majority of patients receiving ranibizumab avoided moderate visual loss (94.6% of those receiving 0.5 mg), while only 62.2% of the control group managed to do so.

In the ANCHOR (Anti-VEGF Antibody for the Treatment of Predominantly Classic Choroidal Neovascularization in AMD) trial, patients with “predominantly classic” angiographic leakage patterns were randomized to receive either 24 monthly intravitreal injections of ranibizumab (either 0.3 mg or 0.5 mg) or photodynamic therapy with verteporfin [79]. Visual acuity improved by 15 letters or more in 40.3% of the 0.5 mg group (as compared with 5.6% of the verteporfin group). As in the MARINA trial, those receiving ranibizumab also demonstrated a mean increase in visual acuity (11.3 letters in the 0.5 mg group), while those in the control group experienced a mean decrease (9.5 letters in the verteporfin group). Similarly, 96.4% of those given 0.5 mg avoided further moderate visual loss (as compared with 64.3% of those in the verteporfin group).

In the two-year HORIZON extension trial of the MARINA and ANCHOR studies, 69% of patients still required further intravitreal injections—despite receiving monthly injections for a two-year period prior to this. Furthermore, the median visual gain at the conclusion of MARINA and ANCHOR decreased with less frequent ranibizumab dosing in the HORIZON trial [81]. 

In the MARINA and ANCHOR trials, the incidence of serious adverse effects was similar between treatment and control groups—for arterial thromboembolic events (e.g., nonfatal myocardial infarctions, nonfatal stroke, and death from a vascular or unknown cause) were 3.8% (sham) versus 4.6% (0.5 mg ranibizumab) in MARINA and 4.2% (PDT) versus 5.0% (0.5 mg ranibizumab) in ANCHOR. An interim analysis from the SAILOR (Safety Assessment of Intravitreal Lucentis for AMD) study showed a trend for an increase in the incidence of stroke in the group treated with 0.5 mg of ranibizumab [82, 83]. Moreover, the incidence of stroke was higher with preexisting risk factors, in particular a previous history of stroke or arrhythmia. These findings, and recent studies reporting an association between AMD itself and higher risk of stroke, suggest that extra discussion of risk-to-benefit ratios may be warranted prior to ranibizumab treatment in patients with a history of stroke [82, 84].

In 2011, the results of the CATT study confirmed the efficacy of ranibizumab in the treatment of neovascular AMD and allowed comparison of outcomes with bevacizumab [61]. In this trial, 34.2% of patients treated with ranibizumab on a fixed, monthly regimen gained 15 or more letters from baseline visual acuity (24.9% for patients treated with ranibizumab as required). In addition, 94.4% of patients receiving ranibizumab on a fixed, monthly regimen lost fewer than 15 letters of visual acuity from baseline (95.4% in the ranibizumab as required group). Finally, mean visual acuity increased by 8.5 letters over the study period in those receiving ranibizumab monthly (6.8 letters in ranibizumab as required group). No statistically significant difference in visual outcomes was found between ranibizumab administered monthly and bevacizumab administered monthly (nor for either drug administered as required). Rates of death, myocardial infarction, and stroke were also similar for patients receiving either ranibizumab or bevacizumab.

### 6.5. Selected Clinical Studies: DME

The use of intravitreal ranibizumab for the treatment of DME has recently been evaluated in a number of phase III clinical trials, including the DRCR (Diabetic Retinopathy Clinical Research) Network and RESTORE studies [85, 86]. In the DRCR Net trial, the mean change in visual acuity from baseline, after one year, was significantly greater in those receiving ranibizumab (+9 letters in those receiving ranibizumab with deferred laser) versus those receiving laser alone (+3 letters) [85]. Similarly, in the RESTORE study, ranibizumab monotherapy resulted in a greater mean change in visual acuity from baseline (+6.1 letters) versus laser monotherapy (+0.8 letters) [86]. In both studies, ranibizumab therapy had a similar safety profile for the treatment of DME as has been previously described for neovascular AMD.

### 6.6. Selected Clinical Studies: CRVO

Treatment of CRVO-associated macular edema was evaluated in the phase III, CRUISE (Central Retinal Vein OcclUsIon Study: Evaluation of Efficacy and Safety) trial [87]. The study was a 6-month, multicenter, randomized, sham injection-controlled study, with an additional 6 months of follow-up (total 12 months). The study included a six-month treatment period, during which subjects received monthly intraocular injections of 0.3 mg or 0.5 mg ranibizumab, or sham injections; and a six-month observation period, during which all patients could receive monthly ranibizumab retreatment if they met prespecified functional and anatomic criteria.

Mean change from baseline visual acuity at month six was +14.9 letters in the 0.5 mg ranibizumab group and +0.8 letters in the sham group. The percentage of patients who gained ≥15 letters in visual acuity at month six were 47.7% in the 0.5 mg ranibizumab and 16.9% in the sham group. In addition, the safety profile was consistent with previous phase III clinical trials of ranibizumab for other ocular disorders, and no new safety events were identified in patients with CRVO. Treatment with ranibizumab as needed, during the six-month observation period, maintained the visual benefits gained in the initial treatment phase [88].

### 6.7. Selected Clinical Studies: Branch Retinal Vein Occlusion

Treatment of branch retinal vein occlusion- (BRVO-) associated macular edema was evaluated in the phase III, BRAVO (B*RA*nch Retinal *V*ein *O*cclusion: Evaluation of Efficacy and Safety) trial [89]. As with the CRUISE study, the BRAVO trial was a 6-month, multicenter, randomized, sham injection-controlled study, with an additional 6 months of follow-up (total 12 months). The study included a six-month treatment period, during which subjects received monthly intraocular injections of 0.3 mg or 0.5 mg ranibizumab, or sham injections; and a six-month observation period, during which all patients could receive monthly ranibizumab retreatment if they met prespecified criteria.

Mean change from baseline visual acuity at month six was +18.3 letters in the 0.5 mg ranibizumab group, and +7.3 letters in the sham group. The percentage of patients who gained ≥15 letters in visual acuity at month six were 61.1% in the 0.5 mg ranibizumab group, and 28.8% in the sham group. As in the CRUISE study, the safety profile of ranibizumab appeared similar to that previous phase III clinical trials of ranibizumab in other disorders. Treatment with ranibizumab as needed, during the six-month observation period, maintained the visual benefits gained in the initial treatment phase [90].

## 7. Aflibercept

VEGF Trap (aflibercept) is a pharmacologically engineered (fusion) protein that blocks the effects of VEGF by acting as a decoy receptor [91]. VEGF Trap consists of the ligand-binding elements of VEGFR1 and VEGFR2, fused to the Fc (fragment crystallizable) portion of human immunoglobulin G1 (IgG1). VEGF Trap is in commercial development by Regeneron Pharmaceuticals Inc. (Tarrytown, NY) in the USA, and by Bayer Healthcare (Leverkusen, Germany) for global markets. The ocular formulation of VEGF Trap is known as “VEGF Trap-Eye” (known generically as aflibercept ophthalmic solution, and to be marketed as “EYLEA”)—an iso-osmotic, ultrapurified formulation for intravitreal injection. An intravenous formulation is also being developed for oncological use (this formulation is hyperosmotic and diluted prior to intravenous infusion) [91]. 

### 7.1. Chemistry

VEGF Trap was first described in 2002 [92]. Prior to this, the highest potency VEGF blocker consisted of a soluble decoy receptor created by fusing the first three Ig domains of VEGFR1 to the constant region (Fc portion) of human IgG1 [91, 93]. This compound demonstrated not only considerable VEGF binding efficacy but also a number of unfavorable molecular and pharmacokinetic characteristics (e.g., nonspecific binding of extracellular matrix components, and the need for frequent administration at high concentrations to achieve efficacious levels in rodent models). Therefore, in its current construct, VEGF Trap consists of the second Ig domain of VEGFR1 and the third Ig domain of VEGFR2, bound to the Fc portion of human IgG1.

VEGF Trap is produced in Chinese hamster ovary cells using recombinant techniques, has a protein molecular weight of 97 kDa, and is approximately 15% glycosylated to yield a total molecular weight of 115 kDa (by comparison, ranibizumab and bevacizumab have protein molecular weights of 48 kDa and 149 kDa, resp.) [91, 94]. VEGF Trap binds VEGF with higher affinity than monoclonal antibodies such as bevacizumab; VEGF Trap may, thus, be active at lower concentrations than other VEGF blocking drugs and may offer a longer duration between doses when compared to other drugs. VEGF Trap is also distinguished from ranibizumab by its ability to bind other VEGF family members—in particular, placental growth factors 1 and 2 (PLGF1 and PLGF2). Finally, the Fc portion of VEGF Trap slows clearance by conferring the long-circulating half-life typically seen with antibodies and, because it contains only human sequences, its potential for immunogenicity is low [92].

### 7.2. Preclinical Studies

The efficacy of VEGF Trap for the suppression of choroidal neovascularization has been evaluated in a number of animal models. In mice, subcutaneous injections, or a single intravitreal injection, of VEGF Trap strongly suppressed choroidal neovascularization following laser-induced rupture of Bruch's membrane [95]. Subcutaneous injection of VEGF Trap has also been shown to significantly inhibit subretinal neovascularization in transgenic mice that express VEGF in their photoreceptors [95]. In rats, VEGF Trap has also been found to inhibit CNV and associated inflammation and fibrosis in a subretinal Matrigel CNV model (Matrigel is a growth factor-reduced, synthetic matrix that has recently been shown to induce CNV formation when injected into the subretinal space of rats) [96]. Finally, in nonhuman primates (adult cynomolgus monkeys), a single intravitreal injection of VEGF Trap has also been shown to induce inhibition of active CNV leakage following laser-induced rupture of Bruch's membrane [94]. 

The efficacy of VEGF Trap in the prevention of blood-retinal barrier breakdown has also been assessed in a number of animal models (i.e., a measure of potential efficacy in retinal vascular diseases such as diabetic retinopathy and retinal venous occlusion) [95]. Following injection of recombinant VEGF into the vitreous cavity of mice, VEGF Trap significantly reduced breakdown of the blood-retinal barrier. Similarly, in transgenic mice where VEGF expression is induced in the retina, VEGF Trap also reduced breakdown of the blood retinal barrier.

### 7.3. Pharmacokinetics and Metabolism

VEGF Trap is cleared from the circulation through one of two pathways: (1) by binding to VEGF to form an inactive complex or (2) by Fc-receptor or pinocytic mediated pathways that culminate in proteolysis [91]. At low blood levels, the clearance of VEGF Trap is rapid as a result of binding to VEGF; at very high circulating doses, VEGF Trap has a terminal half-life of approximately 17 days. The terminal half-life after human intravitreal injection is unknown.

### 7.4. Selected Clinical Studies: Neovascular AMD

The use of intravitreal VEGF Trap-Eye for the treatment of neovascular AMD has recently been evaluated in two phase III clinical trials: the North American VIEW 1 study and the international VIEW 2 study (VIEW: “VEGF Trap-Eye: Investigation of Efficacy and Safety in Wet AMD”). In the VIEW 1 study, patients receiving VEGF Trap-Eye (2 mg), with fixed monthly retreatment, achieved a significantly greater mean improvement in visual acuity at one year (+10.9 letters) compared to patients receiving ranibizumab (0.5 mg) on a similar regimen (+8.1 letters) (however, all other dose groups of VEGF Trap-Eye in the VIEW 1 study, and all dose groups in the VIEW 2 study, were not statistically different from ranibizumab with regard to this endpoint). Of note, subjects receiving VEGF Trap-Eye (2 mg)—every two months—gained, on average, 7.9 letters in VIEW 1 and 8.9 letters in VIEW 2 (http://www.regeneron.com/ accessed August 31, 2011). 

### 7.5. Selected Clinical Studies: CRVO

The use of intravitreal VEGF Trap-Eye for the treatment of central retinal vein occlusion (CRVO) has recently been evaluated in two phase III clinical trials: COPERNICUS (Controlled Phase 3 Evaluation of Repeated intravitreal administration of VEGF Trap-Eye in Central retinal vein occlusion) and GALILEO (General Assessment Limiting Infiltration of Exudates in central retinal vein Occlusion with VEGF Trap-Eye). On December 20, 2010, the results of the COPERNICUS study were announced. In this trial, 56.1% of patients receiving monthly VEGF Trap-Eye (2 mg) gained at least 15 letters of visual acuity, versus 12.3% of controls. Furthermore, the mean change in visual acuity, from baseline, was +17.3 letters versus −4.0 letters for the sham injection group (http://www.regeneron.com/ accessed August 31, 2011).

## 8. Conclusions and Future Directions

A number of points from this review, with significant clinical implications, are worth highlighting. In terms of vascular development, coverage of new vessels by pericytes appears to be a key event resulting in loss of VEGF dependence; awareness of this finding is important for clinicians as the role of anti-VEGF therapies continues to expand, both within the posterior segment and elsewhere in the eye (e.g., treatment of corneal neovascularization). The development of imaging modalities capable of assessing this cell type, and applicable in a clinical setting, is thus of considerable interest.

In terms of drug development and molecular characteristics, a number of important findings emerge. Firstly, the aptamer pegaptanib, while offering low immunogenicity, excellent safety profiles, and the prospect of selective VEGF inhibition, does not provide the extent of VEGF blockade necessary for optimal clinical outcomes in a variety of ocular diseases. Secondly, the antibody fragment ranibizumab, by blocking all VEGF isoforms, has proven effective in large clinical trials for a variety of ocular diseases (neovascular AMD, DME, RVO), with a good systemic safety profile, but at a high cost. Conversely, bevacizumab, a full-length antibody, offers a low-cost alternative to ranibizumab that has recently been validated in large clinical trials. However, some concerns regarding its use include (1) the longer systemic half-life of a full-length antibody, with the potential for increased systemic adverse events, (2) the potential for the Fc component of a full-length antibody to trigger inflammatory processes, (3) the need for precise compounding of the drug for intraocular administration, and (4) the decreased affinity of bevacizumab for VEGF relative to that of ranibizumab. Nonetheless, the experience of retinal specialists worldwide attests to the profound benefits that patients have received following intravitreal bevacizumab therapy. Finally, VEGF Trap appears to have a higher VEGF binding affinity than bevacizumab and, unlike ranibizumab, can bind other members of the VEGF family (e.g., PLGF). VEGF Trap may, thus, offer a longer duration between doses when compared to ranibizumab and bevacizumab.

### 8.1. Future Directions

The introduction of anti-VEGF therapies represented a significant milestone in the medical treatment of retinal diseases. Progress in this area continues to be made at a rapid pace, with the optimization of treatment dosages (e.g., increased dosages of ranibizumab in the ongoing HARBOR trial) and schedules (e.g., PrONTO and CATT studies) and with the development of new agents aimed at anti-VEGF blockage (e.g., siRNAs) [82, 97, 98]. Use of combination therapies may also provide synergistic benefits including better visual outcomes, reduced frequency of treatments, lower risk of adverse events, and decreased likelihood of “escape” (i.e., the development of alternative pathways by which cells allow themselves to overcome iatrogenic inhibition) [99, 100]. Already, combination approaches have been examined in neovascular AMD (e.g., anti-VEGF therapy in combination with verteporfin photodynamic therapy or macular radiation) and in DME (e.g., anti-VEGF therapy followed by macular laser photocoagulation). In the future however, greater visual gains may require more novel strategies aimed at promotion of RPE and photoreceptor survival. In this regard, a number of therapies using neuroprotective, anti-inflammatory, and visual-cycle targeted strategies are in development, for AMD and for other disorders [101]. 

## 9. Disclosure

Dr Keane has received a proportion of his funding from the Department of Health's NIHR Biomedical Research Centre for Ophthalmology at Moorfields Eye Hospital and UCL Institute of Ophthalmology. The views expressed in the publication are those of the authors and not necessarily those of the Department of Health.

Dr Sadda is a co-inventor of Doheny intellectual property related to optical coherence tomography that has been licensed by Topcon Medical Systems, and is a member of the scientific advisory board for Heidelberg Engineering. Dr Sadda also receives research support from Carl Zeiss Meditec, Optos, and Optovue, Inc. 

## Abbreviations

VEGF: Vascular endothelial growth factor
RVO: Retinal vein occlusion
AMD: Age-related macular degeneration
ECM: Extracellular matrix
bFGF: Basic fibroblast growth factor
TGF-*β*: Transforming growth factor-*β*
VPF: Vascular permeability factor
PLGF: Placental growth factor
HIF-1: Hypoxia inducible factor-1
VHL: Von Hippel Lindau
VEGFR1: VEGF Receptor 1
VEGFR2: VEGF Receptor 2
NRP1: Neuropilin 1
ICAM-1: Intracellular adhesion molecule 1
CRVO: Central retinal vein occlusion
siRNA: Small interfering RNA
SELEX: Systematic evolution of ligands by exponential enrichment
PEG: Polyethylene glycol
FDA: Food and Drug Administration
DME: Diabetic macular edema
CDR: Complementarity determining region
RPE: Retinal pigment epithelium
HUVEC: Human umbilical vascular endothelial cell
Fab: Fragment antigen binding
BRVO: Branch retinal vein occlusion
Fc: Fragment crystallizable
IgG1: Human immunoglobulin G1.

## Clinical Trial Acronyms

VISION: VEGF Inhibition Study in Ocular Neovascularization
ABC: Avastin (Bevacizumab) for treatment of Choroidal Neovascularization
CATT: Comparison of Age-Related Macular Degeneration Treatments Trials
MARINA: Minimally Classic/Occult Trial of the Anti-VEGF Antibody Ranibizumab in the Treatment of Neovascular AMD
ANCHOR: Anti-VEGF Antibody for the Treatment of Predominantly Classic Choroidal Neovascularization in AMD
SAILOR: Safety Assessment of Intravitreal Lucentis for AMD
CRUISE: Central Retinal Vein OcclUsIon Study: Evaluation of Efficacy and Safety
BRAVO: B*RA*nch Retinal *V*ein *O*cclusion: Evaluation of Efficacy and Safety
VIEW: VEGF Trap-Eye: Investigation of Efficacy and Safety in Wet AMD
COPERNICUS: Controlled Phase 3 Evaluation of Repeated intravitreal administration of VEGF Trap-Eye in Central retinal vein occlusion
GALILEO: General Assessment Limiting Infiltration of Exudates in central retinal vein Occlusion with VEGF Trap-Eye.
